# Use of Diabetes Treatment Satisfaction Questionnaire in Diabetes Care: Importance of Patient-Reported Outcomes

**DOI:** 10.3390/ijerph15050947

**Published:** 2018-05-09

**Authors:** Yoshifumi Saisho

**Affiliations:** Department of Internal Medicine, Keio University School of Medicine, 1608582 Tokyo, Japan; ysaisho@z5.keio.jp; Tel.: +81-3-5363-3797

**Keywords:** patient-reported outcome, treatment satisfaction, quality of life, ceiling effect

## Abstract

The efficacy of diabetes treatment should not be evaluated solely by HbA1c levels as they should also focus on patient-reported outcomes (PROs), such as patient satisfaction, wellbeing and quality of life. The Diabetes Treatment Satisfaction Questionnaire (DTSQ) has been developed to assess patient satisfaction with diabetes treatment. DTSQ has been translated into more than 100 languages and is widely used in many countries, since it is relatively easy to answer and is used for both patients with and without medical therapy. Novel therapeutic options, such as insulin analogs, incretin-based therapy and sodium-glucose cotransporter 2 (SGLT2) inhibitors, have been shown to improve patient satisfaction using DTSQ for assessments. DTSQ is not only used for comparisons between different medications or treatment strategies, but also can be used to assess the quality of diabetes care in clinical settings. This is important as an improvement in treatment satisfaction may enhance patients’ self-efficacy and adherence to therapy, leading to the achievement of long-term stable glycemic control and reduced risk of diabetic complications. In this review, we summarize the current topics in DTSQ, introducing our own experience, and discuss the role of PROs in diabetes treatment.

## 1. Introduction

The goal of diabetes treatment is the prevention of the onset and progression of micro- and macrovascular complications as well as the achievement of quality of life (QOL) and longevity equivalent to people without diabetes. The UK Prospective Diabetes Study (UKPDS) [1], Diabetes Control and Complications Trial/Epidemiology of Diabetes Interventions and Complications (DCCT/EDIC) [2] and Kumamoto Study [3] have established the importance of glycemic control to prevent diabetic complications and based on this evidence, a glycated hemoglobin (HbA1c) level of <7% is currently recommended as the glycemic goal in most guidelines [4,5].

However, the outcome of diabetes treatments should not be evaluated only by HbA1c levels as the evaluation of the psychological aspects of patients, including treatment satisfaction, wellbeing and quality of life (QOL), are also important, which are referred to as patient-reported outcomes (PROs) (Table 1) [6,7,8,9]. The Diabetes Treatment Satisfaction Questionnaire (DTSQ) is a questionnaire used to assess patients’ satisfaction with their diabetes treatment [10]. DTSQ is now translated into more than 100 languages, including Japanese [11], and is one of the most widely used questionnaires in the field of diabetes. In this review, we summarize the significance and the limitations of the use of DTSQ for the assessment of diabetes treatment and discuss the role of treatment satisfaction in diabetes care.

## 2. DTSQ: Diabetes Treatment Satisfaction Questionnaire

DTSQ was developed by Clare Bradley, an English health psychologist, in the 1990s for the purpose of assessing patients’ satisfaction with their diabetes treatment [10]. It is composed of eight questions, each of which is scored by patients on a scale ranging from zero (e.g., “very dissatisfied”, “very inconvenient”) to six (e.g., “very satisfied”, “very convenient”). The questionnaire is composed of two different factors. The first factor assesses treatment satisfaction and consists of six questions (Q 1, 4, 5, 6, 7 and 8). These six questions ask about “satisfaction with current treatment”, “flexibility”, “convenience”, “understanding of diabetes”, “recommend treatment to others” and “willingness to continue”, respectively. These six questions showed good internal consistency, with a Cronbach alpha score of 0.90 [11]. The second factor consists of two questions (Q 2 and 3), which assess the burden from hyper- and hypoglycemia, respectively (zero being “none of the time” to six being “most of the time”). Treatment satisfaction is assessed as the sum of the scores of the six questions on the first factor (total score 36), with a higher score indicating higher treatment satisfaction.

DTSQ has been translated into more than 100 languages and is widely used in many countries, since it is internationally validated and officially approved by WHO and the International Diabetes Federation (IDF) [10]. The other reasons for this wide usage of DTSQ include the following: (1) it is able to assess treatment satisfaction irrespective of the treatment methods used, including dietary therapy, therapy with oral hypoglycemic agents (OHAs) or insulin therapy; (2) it is relatively easy to answer and places a smaller burden on patients, since the questions are simple and there are only eight items, which is fewer compared to other questionnaires; and (3) the results can be directly compared to those obtained in other countries internationally.

## 3. Significance of DTSQ in Diabetes Treatment

One of the best examples showing the efficacy of DTSQ is the assessment of insulin analogs. Rapid-acting insulin analogs have been shown to improve postprandial glycemic excursion and reduce hypoglycemia compared with regular insulin due to their rapid onset of action [12]. They also allow patients to inject at mealtimes, which promotes convenience when compared with regular insulin, which requires injection 30 mins before a meal. However, studies have shown the same efficacy of rapid-acting insulin analogs compared with regular insulin in terms of HbA1c levels. In contrast, the comparison using DTSQ has clearly demonstrated the improvement in patients’ satisfaction with rapid-acting insulin analogs compared to regular insulin [13]. Similarly, the improvement in patients’ satisfaction, but not HbA1c levels, has been shown using DTSQ in the patients treated with long-acting insulin analogs compared with those treated with neutral protamine Hagedorn (NPH) insulin [14]. These results emphasize the importance of patient satisfaction in the assessment of diabetes treatment, which cannot be fully assessed by glycemic indices, including HbA1c.

To date, the assessment of PROs, including DTSQ, is essential for evaluating the efficacy of novel anti-diabetic agents and assessing the glucose-lowering effect related to HbA1c levels. The improvement in patient satisfaction has been also shown in the patients treated with incretin-related agents [8,15,16], sodium-glucose cotransporter 2 (SGLT2) inhibitors [17], fixed-dose combination tablets [18] and weekly dipeptidyl peptidase-4 (DPP-4) inhibitors [19].

## 4. DTSQ and Drop Out: Experience in Our Institution

We have evaluated patient satisfaction using DTSQ in outpatients with T2DM in our institution, which is a university hospital in an urban area (*n* = 299). The results have been reported previously. However, although the original articles were written in Japanese, we would like to describe the study briefly [20,21]. We used the Japanese version of DTSQ, which was validated previously [11]. Questionnaires regarding the clinical parameters were also conducted simultaneously and the relationships of these clinical parameters with DTSQ scores were assessed. The questionnaires were filled out anonymously and the patients answered the questionnaires in the waiting room outside the examination room. As a result, the mean total score of DTSQ (the sum of six questions = 0–36) was 25.6 (median = 25 and interquartile range of 21–30), with the mean score of each question being 4.1–4.5. The mean scores of Q 2 (“hyperglycemia”) and 3 (“hypoglycemia”) were 3.0 and 1.4, respectively.

With the exception of Q 2 and 3, the six questions related to the first factor were significantly correlated with each other as well as with the total score (Table 2) [20]. In particular, Q 4 (“flexibility”), 7 (“recommend treatment to others”) and 8 (“willingness to continue”) showed strong correlations with the total score (ρ > 0.8). There was also a significant positive correlation between Q 2 and 3. Q 2 was negatively correlated with Q 1, while Q 3 was negatively correlated with Q 4, 5, 8 and the total score. These results suggest that the patients who experience a greater burden of hyperglycemia also tend to experience a greater burden of hypoglycemia, while the burden of hypoglycemia is more strongly correlated with reduced treatment satisfaction compared to the burden of hyperglycemia.

When the associations between the DTSQ score and other clinical parameters were examined, there was a weak negative correlation between the total score of DTSQ and the intensity of treatment (i.e., dietary therapy only > therapy with OHAs > insulin therapy) (Table 3). This suggests a negative association between treatment burden and treatment satisfaction, although a previous study reported an improvement in the DTSQ score after implementation of insulin therapy in poorly-controlled patients with T2DM [22]. There was no apparent correlation between total DTSQ score and age or sex in our cohort.

On the other hand, we did not observe any significant correlation between HbA1c level and total DTSQ score. No or only modest associations between HbA1c levels and DTSQ score have also been reported [11,13,14], which is consistent with our results. This indicates that treatment satisfaction is not necessarily related to glycemic control.

It is of note that patients who reported that they had better adherence to lifestyle modification (dietary therapy and increased physical activity) and/or medical therapy in the questionnaire showed higher scores in DTSQ (Table 3). This suggests that patients with higher treatment satisfaction also experience higher self-efficacy, resulting in better adherence to therapy. Moreover, in our cohort, the patients who answered yes to the question “Have you ever intended to dropout from therapy?” showed significantly lower total DTSQ scores (21.6 ± 6.9 vs. 26.2 ± 6.2, P = 0.006). The receiver operating characteristic (ROC) analysis revealed that the total score of DTSQ predicted the intention to dropout (area under the curve; AUC = 0.696) with a cut-off value of 22.5 (sensitivity 63.2%, specificity 70.8%) [21]. These results indicate that the assessment of treatment satisfaction with DTSQ may predict dropout from therapy. On the contrary, an improvement in treatment satisfaction, which can be assessed with DTSQ, may reduce the risk of dropout.

In our cohort, the total DTSQ score was also negatively correlated with waiting time in the hospital (Table 3), suggesting that treatment satisfaction assessed with DTSQ is not only affected by treatment itself, but is also affected by other factors, such as waiting time, distance from the hospital and cost. Of note, we found that the satisfaction with their attending doctor showed the strongest association with the total DTSQ score among the clinical parameters. Hence, DTSQ is a powerful tool that can compare treatment satisfaction between different medications or treatment strategies. However, the relationship between a patient and doctors (medical staff), rather than medications or treatment strategies, may have a major impact on treatment satisfaction.

## 5. Limitations of DTSQ

As with other questionnaires, there are limitations of the DTSQ. The “ceiling effect” is a limitation of DTSQ [23]. If DTSQ score at baseline is already high enough, it will be difficult to detect further improvement in treatment satisfaction after intervention. On the other hand, if the scores of Q2 (“hyperglycemia”) and Q 3 (“hypoglycemia”) are already low enough at baseline, it will also be difficult to detect an improvement after intervention, which is referred to as the “floor effect”. To overcome this issue, DTSQ changed version (DTSQc) has been proposed [23], in which the patients are asked to consider their satisfaction with their current treatment compared with their previous treatment, which aims to more effectively assess change after interventions.

The results of DTSQ are not limited to a specific medication or treatment strategy, but rather the overall satisfaction with the treatment of diabetes. As discussed above, DTSQ scores may also be affected by various factors, including waiting time and satisfaction with consultation with medical staff. Therefore, DTSQ scores assessed in different institutions with different medical staff or under different conditions may not be directly comparable and thus, caution is needed when interpreting the results.

Finally, DTSQ is a tool to assess treatment satisfaction that is specifically related to diabetes. Although the improvement in treatment satisfaction assessed with DTSQ is expected to improve patients’ QOL, it should be stressed that DTSQ does not assess QOL itself. Indeed, as QOL of patients with diabetes has been shown to be lower, a higher DTSQ score does not necessarily translate into higher QOL [6]. Thus, the use of questionnaires other than DTSQ, such as SF-36 and WHOQOL, is needed to evaluate QOL. Furthermore, as co-morbidities may affect the results of DTSQ, the use of more than two different measures should be considered to comprehensively evaluate PROs.

**Figure 1:**
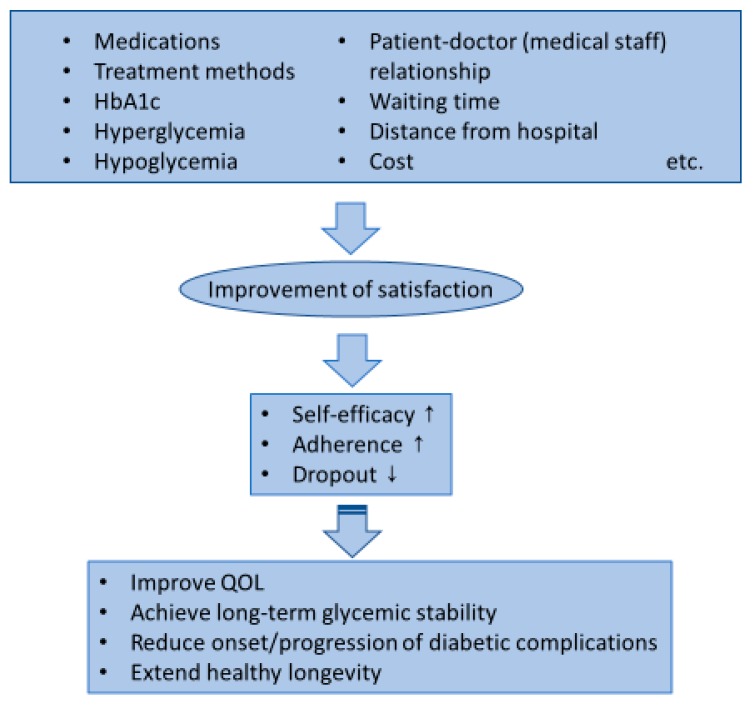
Factors associated with treatment satisfaction and expected effects from its improvement on clinical outcomes.

It also remains unclear whether an improvement in the DTSQ score translates into an improvement in other clinical outcomes, including cardiovascular outcomes and overall mortality. Since the DTSQ score predicted dropout from treatment, the improvement in DTSQ score may improve patients’ adherence to treatment and reduce the risk of dropout. Although there has been no prospective study addressing this question to date, reducing the incidence of dropout by improving treatment satisfaction may promote long-term glycemic stability and reduce the risk of diabetic complications (Figure 1).

## 6. Conclusions

DTSQ is a questionnaire to evaluate patients’ satisfaction with their diabetes treatment, which is now translated into more than 100 languages and widely used in many countries. The efficacy of diabetes treatment should not be evaluated solely by HbA1c levels. The improvement in the treatment satisfaction results in improving patients’ self-efficacy and adherence to treatment. The improvement in treatment satisfaction may also reduce the risk of dropout from treatment. Therefore, improvement in treatment satisfaction may foster the achievement of long-term glycemic stability, eventually reducing the risk of developing diabetic complications (Figure 1). The assessment of treatment satisfaction is not only performed for research purposes to compare treatments, but also should be used to assess the quality of diabetes care in clinical settings. A better understanding of the role of PROs in diabetes care and appropriate use of each questionnaire, including DTSQ, is needed for further improvement of current diabetes treatment.

## Figures and Tables

**Table 1:** Major questionnaires for PROs used internationally in patients with diabetes. This table was modified and adopted from reference [7].

Name	Number of Items	Subjects (Generic/Diabetes-Specific)	Outcome
ADDQOL	20	Diabetes-specific	QOL
BDI	21	Generic	Wellbeing
DQOL	46	Diabetes-specific	QOL
DTR-QOL	29	Diabetes-specific	QOL
DTSQ	8	Diabetes-specific	Satisfaction
EQ-5D	6	Generic	Health status
HADS	14	Generic	Wellbeing
OHA-Q	20	Diabetes-specific	Satisfaction
PAID	20	Diabetes-specific	Wellbeing
SF-36	36	Generic	Health status
W-BQ	12 28	Generic Generic/diabetes-specific	Wellbeing
WHOQOL	100/26	Generic	QOL

ADDQOL = Audit of Diabetes-Dependent Quality of Life; BDI = Beck Depression Inventory; DQOL = Diabetes Quality of Life; DTR-QOL = Diabetes Therapy-Related QOL; DTSQ = Diabetes Treatment Satisfaction Questionnaire; EQ-5D = Euro-QoL 5-Dimension; HADS = Hospital Anxiety and Depression Scale; OHA-Q = Oral Hypoglycemic Agent Questionnaire; PAID = Problem Areas in Diabetes; SF-36 = Short-Form 36; W-BQ = Well-Being Questionnaire; and WHOQOL = World Health Organization Quality of Life.

**Table 2:** Correlations between each question (Q 1 to 8) and the total DTSQ scores. This was modified and adopted from reference [20].

Item	Q 1	Q 2	Q 3	Q 4	Q 5	Q 6	Q 7	Q 8	Total
Q 1	-	−0.133 *	−0.104	0.640 *	0.522 *	0.490 *	0.568 *	0.681 *	0.776 *
Q 2	−0.133 *	-	0.215 *	−0.001	0.001	−0.069	−0.035	−0.095	−0.076
Q 3	−0.104	0.215 *	-	−0.176 *	−0.151 *	−0.078	−0.114	−0.205 *	−0.175 *
Q 4	0.640 *	−0.001	−0.176 *	-	0.734 *	0.509 *	0.645 *	0.705 *	0.870 *
Q 5	0.522 *	0.001	−0.151 *	0.734 *	-	0.412 *	0.594 *	0.633 *	0.797 *
Q 6	0.490 *	−0.069	−0.078	0.509 *	0.412 *	-	0.579 *	0.557 *	0.710 *
Q 7	0.568 *	−0.035	−0.114	0.645 *	0.594 *	0.579 *	-	0.708 *	0.838 *
Q 8	0.681 *	−0.095	−0.205 *	0.705 *	0.633 *	0.557 *	0.708 *	-	0.868 *
Total	0.776 *	−0.076	−0.175 *	0.870 *	0.797 *	0.710 *	0.838 *	0.868 *	-

Total score was calculated as the sum of scores of Q 1, 4, 5, 6, 7 and 8. Q 1 = “satisfaction with current treatment”; Q 2 = “hyperglycemia”; Q 3 = “hypoglycemia”; Q 4 = “flexibility”; Q 5 = “convenience”; Q 6 = “understanding of diabetes”; Q 7 = “recommend treatment to others”; and Q 8 = “willingness to continue”. The values are Spearman’s rank correlation coefficients. * *p* <0.05.

**Table 3:** Correlations between total DTSQ score and clinical parameters. This was modified and adopted from reference [20].

Clinical Parameters	ρ
Sex	0.035
Age	0.073
Duration of diabetes	0.034
Therapy (dietary/OHA/insulin)	−0.120 *
HbA1c	0.024
Adherence to dietary therapy	0.270 *
Adherence to increased physical activity	0.224 *
Adherence to medication	0.363 *
Satisfaction with waiting time	−0.189 *
Satisfaction with consultation time	0.177 *
Satisfaction with attending doctor	0.504 *
Satisfaction with overall hospital visit	0.462 *
History of dropout	0.009
Intention to dropout	−0.203 *

The total DTSQ score was calculated as the sum of scores of Q 1, 4, 5, 6, 7 and 8. The values are Spearman’s rank correlation coefficients. * *p* <0.05. OHA = oral hypoglycemic agents.
